# Idiopathic Anaphylaxis: a Perplexing Diagnostic Challenge for Allergists

**DOI:** 10.1007/s11882-021-00988-y

**Published:** 2021-02-09

**Authors:** Theo Gulen, Cem Akin

**Affiliations:** 1 Department of Respiratory Medicine and Allergy, K85, Karolinska University Hospital, Huddinge, SE-141 86 Stockholm, Sweden; 2 Immunology and Allergy Unit, Department of Medicine Solna, Karolinska Institutet and Karolinska University Hospital, Stockholm, Sweden; 3 Mastocytosis Center Karolinska, Karolinska Institutet and Karolinska University Hospital, Stockholm, Sweden; 4 Division of Allergy and Clinical Immunology, Department of Internal Medicine, University of Michigan Health System, Ann Arbor, MI USA

**Keywords:** Anaphylaxis, Mast cells, Idiopathic anaphylaxis, Tryptase, Mast cell activation, Mastocytosis

## Abstract

**Purpose of Review:**

The aim of this systematic review is to present the proposed theories of pathogenesis for idiopathic anaphylaxis (IA), to discuss its classification, its diagnostic approach, and management.

**Recent Findings:**

IA represents a major diagnostic challenge and is diagnosed when excluding the possible identifiable triggers of anaphylaxis. The current research, however, revealed that certain conditions including mastocytosis, mast cell activation syndromes, and hereditary alpha tryptasemia can masquerade and overlap with its symptomatology. Also, newly identified galactose-alpha-1,3-galactose mammalian red meat allergy has recently been recognized as underlying cause of anaphylaxis in some cases that were previously considered as IA.

**Summary:**

IA comprises a heterogenous group of conditions where, in some cases, inherently dysfunctional mast cells play a role in pathogenesis. The standard trigger avoidance strategies are ineffective, and episodes are unpredictable. Therefore, prompt recognition and treatment as well as prophylaxis are critical. The patients should always carry an epinephrine autoinjector.

## Introduction

Mast cells (MCs) are granulated, tissue-dwelling multifunctional effector cells that are normally found in almost all tissues [1–3]. MCs constitutively display a range of biologically active receptors including high-affinity receptors for IgE, (FcϵRI), and surface G protein-coupled receptors including recently reported Mas-related G protein receptor (MRGPRX2), which can be activated through non-IgE pathways by small molecules, including certain drugs [4–7]. Upon activation, these cells degranulate and release a large array of pro-inflammatory and vasoactive mediators and signaling molecules including histamine, prostaglandins, and leukotrienes [7, 8].

Anaphylaxis is the most severe systemic hypersensitivity reaction, and MCs appear to be the primary effector cells for driving anaphylaxis in humans as increased levels of mast cell mediators such as tryptase and histamine have been detectable during episodes. Additionally, basophils may theoretically contribute to symptom development, when activated, by secreting mediators including histamine and LTC_4_. To date, no specific biomarker has been identified to predict patients at risk, to stratify severity of reactions or to optimize management.

Food, drugs, and stinging insects represent the most commonly identified triggers of anaphylaxis. However, in certain patients with anaphylaxis, no eliciting factor can be identified despite a comprehensive allergy workup, and therefore, the term “idiopathic anaphylaxis” (IA) was introduced. IA was first described by Bacal and colleagues in 1978 in a report of 11 patients whose episodes could not be explained by a known trigger [9]. IA is a phenotype of anaphylaxis, and clinically, the presentation is similar to that of other types of anaphylaxis. It is caused by recurrent episodes of mediator-related multiorgan symptoms (at least 2 organ involvement) presenting with various combinations of urticaria, angioedema, laryngeal tightness, bronchoconstriction, dyspnea, hypoxia, abdominal pain, nausea, vomiting, diarrhea, hypotension, and syncope [10]. In rare cases, the IA episodes may lead to death due to the cardiovascular collapse.

The recent research exposed that IA can masquerade as an underlying mast cell dysfunction leading to uncontrolled mast cell activation, presumably by lowered mediator release threshold [11••, 12, 13•]. As our understanding of mast cell disorders continues to grow, the classification for these disorders evolves. The purpose of this article is, therefore, to discuss the pathogenesis of IA within the broader context of mast cell activation disorders as wells as reviewing its epidemiology, clinical manifestations, and diagnosis.

## Epidemiology

The available epidemiological data about the exact prevalence and incidence of anaphylaxis are limited and often inconsistent. However, it is widely accepted that anaphylaxis is a relatively rare condition. With these limitations, the lifetime prevalence of anaphylaxis in the general population has been estimated to be between 0.3 and 1.6% [14, 15].

Nevertheless, the actual incidence and prevalence of IA are difficult to estimate, since its prevalence varied among different reports. This is probably due to using different definitions or diagnostic criteria (not applying consensus criteria before 2006), diagnostic limitations in older studies, for instance overlooked mast cell disorders, and possible over-diagnosing of IA by emergency departments. However, one indirect approach to estimate its incidence is to use the percentage of anaphylaxis cases presenting to an allergist/immunologist that remained idiopathic despite extensive evaluation [16]. By this means, between 20,592 and 47,024 total cases of IA have been reported in the USA, thereby the prevalence was estimated approximately 1 in 10,000 in the mid-1990s [16, 17]. Consequently, approximately 30–60% of cases of anaphylaxis in adults and 10% of cases in children are deemed idiopathic after an extensive evaluation [18–20]. Additionally, there is a higher incidence of IA in women than in men and a higher incidence in adults than in children [20–22]. It was also reported that patients with IA also have a high rate of atopy, approximately 50% [21, 23]. Consequently, larger populations studies are needed to determine the true prevalence, incidence, and demographics of IA.

## Pathogenesis

The underlying mechanisms leading to idiopathic anaphylaxis are not fully understood, although various theories were proposed to explain pathogenesis. One possible theory for IA involves elevated numbers of activated lymphocytes. IA patients have been shown to have a higher percentage of activated T cells (defined as CD3^+^ HLA-DR^+^) in their blood during acute episodes compared to baseline levels [24]. Moreover, IA patients also have more activated B cells (CD19^+^ CD23^+^) during both acute episodes and at baseline compared to control patients or patients with chronic idiopathic urticaria [24]. Interestingly, another study reported that soluble CD25 levels were higher in patients with anaphylaxis after an acute episode compared to healthy controls but were lower than mastocytosis patients [25]. Although this may provide further support for T cell activation involvement in anaphylaxis process, the role played by activated T- and/or B-lymphocytes in the pathogenesis of IA or interaction of these cells with MC and basophils remains elusive. Additionally, due to the fact that females are more often diagnosed with IA, a potential effect of female hormones on MC or basophils has been proposed [26, 27]. Nonetheless, no difference has been shown regarding basophil activation in IA patients compared to controls. Gene expression profiles in patients with IA were compared to nonatopic controls by DNA microarray analyses in a study which found that cells from patients with IA differentially expressed genes correlating with the level of CD203c, and among these genes, some were involved in regulation of mast cell/basophil degranulation [28].

Another attractive theory of IA pathogenesis suggests increased activation of mast cells in IA patients. This is due to the hypothesis that IA patients have hyperreactive mast cells that are prone to degranulation because of the presence of extracellular Th2 cytokines [17]. This hypothesis was supported by the findings that IA patients were found to have increased levels of IL-4, IL-5, and IL-13 cytokines after lymphocyte stimulation compared to atopic subjects and healthy controls [29]. However, in a prospective study of patients with IA, ex vivo studies of cultured mast cells indicated no evidence of a hyperreponsive mast cell phenotype, when tested for IgE-mediated release of beta-hexosaminidase [13•]. Nevertheless, other mechanisms have not been specifically studied. Interestingly, the investigators did note that peripheral blood from patients with IA yielded higher mast cell numbers in culture compared to healthy controls [13•] supporting the notion that MCs have multiple other mechanisms for activation.

Moreover, there are further supporting data that IA is as a mast cell activation disorder [30–33]. First, the predominant cell type known to cause anaphylaxis in humans is the mast cell. It should be noted that although non-mast-cell-dependent anaphylaxis has been reported in mice and is thought to be caused by activation of myeloid cells such as neutrophils and macrophages [34, 35]. However, to the best of our knowledge, this phenomenon has not been proven in humans. Second, elevated tryptase levels during an acute IA episode support mast cell degranulation [33, 36, 37]. Third, patients improve with mast cell–directed therapy including antihistamines, cysteinyl leukotriene receptor antagonists, and corticosteroids [19, 22, 38]. Therefore, human disorders involving anaphylaxis are thought to be predominantly mast cell dependent.

## Idiopathic Anaphylaxis in the Context of Mast Cell Activation Disorders

The nomenclature and classification of mast cell disorders has evolved during the recent years as our knowledge continuously increased on these disorders. For instance, mastocytosis is a clonal mast cell disorder and previously the proliferation and accumulation of abnormal MC in different organs has been emphasized as a typical characteristic of this condition [39, 40]. However, the research during the last decade recognized that activation of MC in mastocytosis is equally important since patients with systemic mastocytosis (SM) have a higher risk for an excessive mediator release leading to severe anaphylaxis [41, 42]. Therefore, a new nomenclature has been proposed to encompass all current diagnoses regarding mast cell disorders and the term mast cell activation syndrome (MCAS) has been introduced [43–45].

Three sets of criteria are required to fulfill MCAS diagnosis: typical episodic symptoms which are attributable to mast cell activation, objective laboratory evidence of mast cell involvement by confirming a substantial transient increase in validated mast cell mediators in the serum (preferably, at least 20% increase in serum tryptase levels plus 2 ng/ml within 4 h of acute episode) or in the urine, and control of symptoms with mast cell–directed therapies [45]. Once a diagnosis of MCAS has been confirmed, further classification is necessary. Accordingly, MCAS have been classified into three types. Primary MCAS, that is associated with clonal mast cell disorders, comprise monoclonal MCAS and mastocytosis, which are characterized by underlying intrinsic mast cell defects, such as tyrosine kinase *KIT* mutation (D816V) or alterations in enzymes or expression of aberrant MC receptors including CD25, that, in turn, confer higher risks for uncontrolled mast cell activation. Diagnosis of clonal MCAS can only be made after an extracutaneous biopsy, most often after a bone marrow biopsy [39, 40]. Additionally, clonal MCAS can present with the clinical manifestations of IA [11••, 12, 46].

**Fig. 1:**
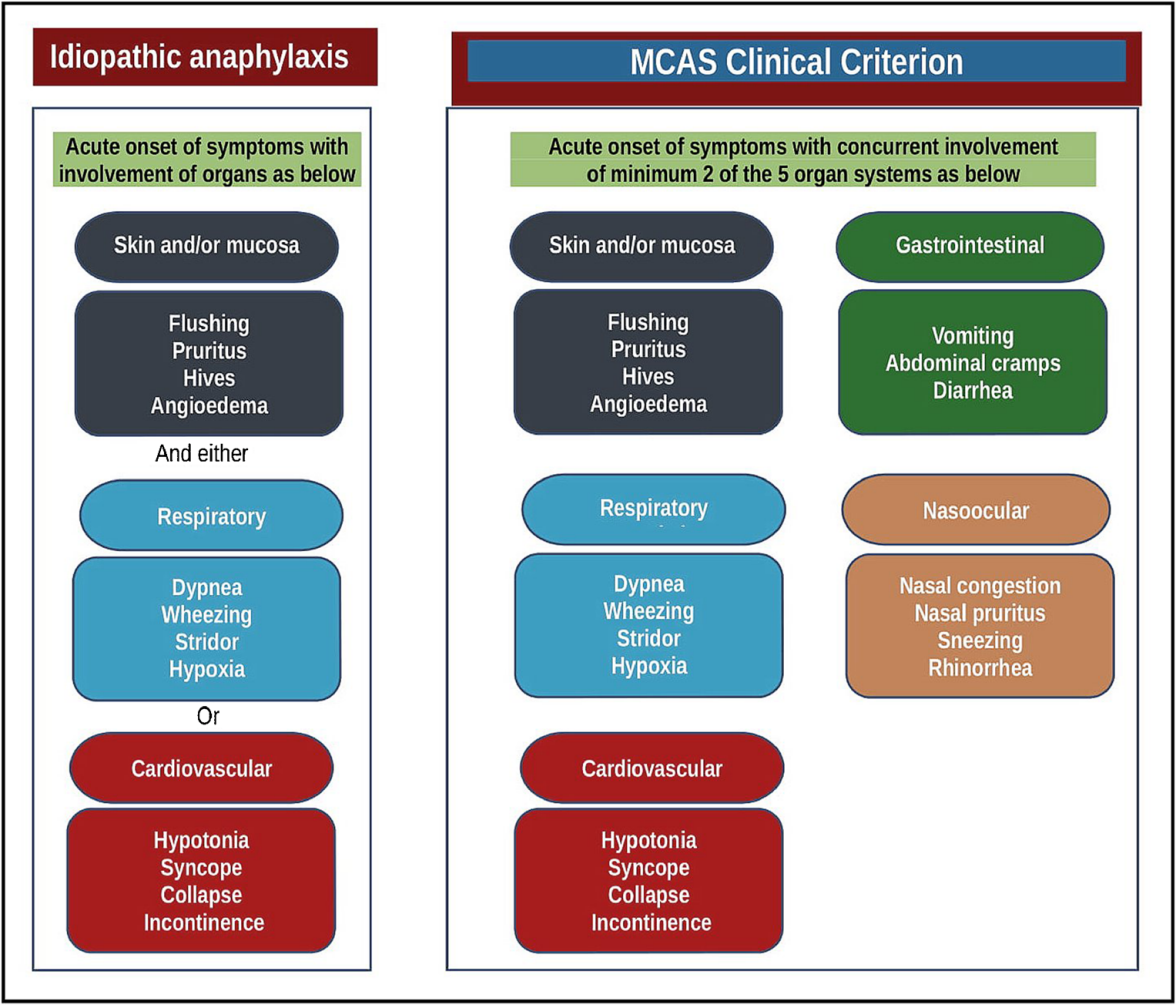
The criteria of idiopathic anaphylaxis versus mast cell activation syndrome. Figure modified from references [10, 40]. Note that when there is no likely cause of the reactions, if the onset of illness is acute, a diagnosis of idiopathic anaphylaxis can only be made when either reduced blood pressure (or its symptoms such as syncope) and/or respiratory compromise are present accompanied by the involvement of the skin–mucosal tissue symptoms. Please see the text for more discussion

Secondary MCAS results in symptoms of mast cell activation through IgE and non-IgE-mediated processes, such as food-, drug-, or *Hymenoptera* venom–induced severe anaphylaxis. Finally, idiopathic MCAS results in mast cell activation symptoms without a clear precipitating etiology. Patients with IA are the epitome of idiopathic MCAS; therefore, it is essential to evaluate whether the patient meets criteria for IA (Fig. 1). However, idiopathic MCAS is a broader entity and may also include patients whose episodes may not fulfill the clinical criteria of idiopathic anaphylaxis, such as patients presenting with concomitant skin and gastrointestinal symptoms.

## Clinical Criteria for Anaphylaxis

Lack of globally recognized definition of anaphylaxis has long caused failure to diagnose and delayed treatment in patients and also hampered research efforts. Subsequently, multinational, multidisciplinary symposia were convened to achieve a true international consensus on the clinical criteria for the diagnosis of anaphylaxis [10].

Signs and symptoms of anaphylaxis are generally related to the cutaneous, gastrointestinal, respiratory, and cardiovascular systems, and the diagnosis requires concurrent occurrence of minimum 2 organ systems. The required organ system involvement also varies depending upon whether there is a “likely” or “known” trigger for the actual patient. For instance, combination of symptoms by the involvement of the skin–mucosal tissue and gastrointestinal tract can be considered anaphylaxis in context of food allergies, if there is a “likely” trigger for the patient. Exceptionally, in context of confirmed allergy, for instance venom or drug, the related patients can develop anaphylaxis only by cardiovascular system involvement (drop in blood pressure or syncope) after re-exposure. Additionally*,* even when the allergic status of the patient is unknown or there is no likely cause of the reactions, as in idiopathic anaphylaxis, when the onset of illness is acute (minutes to several hours), a diagnosis of anaphylaxis can be made when either reduced blood pressure (or associated symptoms of end-organ dysfunction, such as syncope) and/or respiratory compromise or laryngeal edema are present accompanied by the involvement of the skin–mucosal tissue symptoms [10]. Figure 1 illustrates the clinical criteria of both IA and MCAS.

The clinical manifestations of IA were reviewed in a series of 335 patients [19]. Interestingly, all had experienced angioedema and urticaria in this study. In addition, 63% experienced upper airway obstruction, 39% bronchospasm, 23% hypotension or syncope, and 22% gastrointestinal symptoms [19]. Isolated cardiovascular collapse was not reported in these series. Notably, clonal MCAS patients presenting with IA may more prevalently suffer from cardiovascular symptoms, as it was reported to be over 90% in a study [12]. Table 1 demonstrates organ-based symptoms of idiopathic anaphylaxis attributable to release of mast cell mediators.

**Table 1:** Organ-based symptoms of idiopathic anaphylaxis attributable to release of mast cell mediators and potential therapeutics

Clinical features	Attributed mediators	Possible therapeutic interventions
Cardiovascular	Histamine, PAF, PGD2, cysteinyl LTs, TNF, tryptase, renin, chymase	In emergency: intramuscular epinephrine In prevention: H1R- and H2R-antihistamines Antileukotrienes Prednisone Omalizumab
Hypotension		
Syncope		
Tachycardia		
Light-headedness		
Cutaneous	Histamine, PAF, PGD2, cysteinyl LTs, TNF	H1R- and H2R-antihistamines Ketotifen Aspirin or NSAID
Flushing		
Pruritus		
Urticaria		
Angioedema		
Respiratory	Histamine, PGD2, cysteinyl LTs, PAF	Antileukotrienes Inhalation steroids—if asthma diagnosed H1R-antihistamines
Shortness of breath		
Wheezing		
Inspiratory stridor		
Hypoxia		
Digestive	Histamine, PGD2, TNF, cysteinyl LTs, PAF, tryptase, serotonin,	H2R-antihistamines Cromolyn sodium (oral formulation) Glucocorticoids
Abdominal cramps		
Diarrhea		
Vomiting		

*PGD*_*2*_, prostaglandin D_2_; *LT*, leukotrienes; *PAF*, platelet activation factor; *TNF*, tumor necrosis factor; *H1R*, histamine-1-receptor; *H2R*, histamine-2-receptor; *NSAID*, non-steroidal anti-inflammatory drugs

## Diagnostic Approaches and Differential Diagnosis

The differential diagnosis of IA is broad and includes among others all causes of anaphylaxis such as food, hymenoptera venom, drugs, and exercise. Other allergic conditions such as acute urticaria and/or angioedema, acute asthma episodes involve a single organ system but may mimic anaphylaxis. Additionally, some endocrine (such as pheochromocytoma, carcinoid, flushing disorders), cardiovascular (such as postural orthostatic tachycardia syndrome), and psychiatric conditions (such as a panic disorder and somatoform disorder) may present with similar findings, and therefore should be considered in differential diagnosis.

Hidden allergens have been a matter of debate in the etiology of IA [47–49]. However, to the authors’ experience, these are not common causes of IA, but they should be considered in patients, in particular, when an anaphylactic episode was suspected after recent meal by evaluating the ingredients [50•]. We will further review some of the conditions to be considered in the work-up in detail below.

### MCAS and Mastocytosis

As mentioned above, there is an intriguing relationship between IA and MCAS and mastocytosis. Because clonal mast cell disorders can be potentially misdiagnosed as IA, it is, therefore, essential to distinguish them from IA. Akin et al. [11••] reported presence of a clonal MC population in 5 out of 12 patients with IA in whom there were no features of urticaria pigmentosa or histological evidence for SM on bone marrow biopsies performed locally outside of a mast cell disease referral center. Likewise, Gulen et al. [12] performed bone marrow examination in 30 cases of IA with no signs of cutaneous mastocytosis to look for evidence of clonal MC population, and also to try to identify predictive markers that can possibly distinguish between different IA phenotypes. They reported that 47% of IA patients were found to have an aberrant MC population and were subsequently diagnosed with clonal MC disorder. Finally, a recent study investigated 56 subjects with unexplained anaphylaxis, who had more than three episodes/year, with BM examination and found evidence of MC clonal disease in 14% of cases [13•]. The main reason for this discrepancy may be due to the patient selection criteria when diagnosing IA.

There is an unmet need for developing robust criteria to select IA patients for bone-marrow examination in cases of suspected clonal MC disorders. Hence, the search for predictive factors is crucial. In this regard, the Spanish Network on Mastocytosis (Red Española de Mastocitosis [REMA]) proposed a scoring tool [51]. This model is based on a combined clinical (i.e., gender and clinical symptoms) and laboratory (baseline tryptase value with “cut-off” values of < 15 or > 25 ng/mL) criteria, to predict underlying MC clonality in patients presenting with anaphylaxis [51] (Table 2). A modification of the “REMA-score” has been later proposed as the “Karolinska-score,” using a baseline serum tryptase “cut-off” value of 11.4–20 ng/ml [12] (Table 2). This modified version resulted in a better sensitivity (93%) and specificity (94%), when retrospectively applied to their study cohort. Finally, a further improvement of previous tools was proposed, so-called the NIH Idiopathic Clonal Anaphylaxis Score (NICAS), by using clinical symptoms, gender, a baseline tryptase “cut-off” of 11.4 ng/ml, and allele-specific PCR testing to detect the presence or absence of *KIT* D816V mutation in peripheral blood [13•].

**Table 2:** Scoring tools to evaluate IA patients for bone-marrow examination in cases of suspected clonal mast cell disorders

Variables	REMA-score		Karolinska-score	
	Yes	No	Yes	No
Gender				
Male	1	− 1	1	− 1
Clinical symptoms				
Urticaria/Angioedema	− 2	1	− 2	1
Pruritus	− 2	1	− 2	1
Flushing	n/a	n/a	n/a	n/a
Syncope	3	0	3	0
Baseline tryptase				
≤ 11.4 ng/ml	n/a	n/a	−1	n/a
11.4–20 ng/ml	n/a	n/a	0	n/a
> 20 ng/ml	n/a	n/a	2	n/a
< 15 ng/ml	−1	n/a	n/a	n/a
15–25 ng/ml	0	n/a	n/a	n/a
>25 ng/ml	2	n/a	n/a	n/a
Total score*	≥ 2 points		≥ 2 points	
Outcome	High risk		High risk	

Modified from Refs. [12, 51]

*REMA*, Red Española de Mastocitosis; *n/a*, non-applicable

*The sum of positive and negative points of ≥ 2 is considered to be positive and indicates a high probability for underlying clonal MC disorders, thereby warranting a bone marrow examination

*Hereditary alpha-tryptasemia* (HαT) is an autosomal dominant inherited genomic variant of uncertain significance caused by duplication or multiple copy numbers of the α-tryptase gene (TPSAB1) copy number and increased numbers of MCs in bone marrow biopsy specimens [52, 53•] but generally does not have increased urinary secretion of other mast cell mediators. HαT is reported in approximately 6% of the general population, in which sBT levels are typically greater than 8.0 ng/ml [53•, 54]. These patients may present with a clinical phenotype including Ehlers-Danlos syndrome-like symptoms of joint hypermobility with arthritis, postural orthostatic tachycardia syndrome (POTS), flushing or gastrointestinal hypomotility, vibratory urticaria, irritable bowel syndrome, eosinophilic esophagitis, neuropsychiatric diagnoses, chronic musculoskeletal pain, and allergic disorders affecting the cutaneous, respiratory, or cardiovascular systems [52, 53•, 55, 56]; however, this may be subject to referral bias and there has not been consistency in clinical phenotype in unselected cohorts. The etiology of the symptom complex that have been associated with HαT remains unknown. The risk for severe spontaneous or/and insect venom–triggered anaphylaxis episodes in HαT patients was reported to be increased [57–59]. Interestingly, a recent study reported that increased germline copies of α-tryptase are associated with increased severity of venom anaphylaxis and are more prevalent among individuals with idiopathic anaphylaxis and SM, and are associated with an increased relative risk for anaphylaxis among patients with SM [59]. Thus, HαT may confer an increased risk for severe anaphylaxis which is independent of the presence of concomitant clonal mast cell disorders. These findings need to be confirmed in larger patient cohorts, and to date, no studies have shown that mast cells in patients with HαT are hyperresponsive.

### Alpha-gal Syndrome

Recent research has demonstrated an increasing prevalence mammalian of red meat allergy and anaphylaxis triggered by exposure to a mammalian oligosaccharide, galactose-α-1,3-galactose (α-gal) [60•]. Thus, some patients previously carrying the label of IA have now been diagnosed with α-gal syndrome. Unlike other typical IgE-mediated reactions to food, mammalian meat allergy can manifest with symptoms that are delayed up to 6 h after ingestion [60•]. The delayed response is thought to be associated with the digestive process needed to expose the carbohydrate epitope [61]. However, factors such as exercise, alcohol, or aspirin may lower the threshold of responsiveness to α-gal [62]. The time span between food consumption and symptoms likely masked the etiology of anaphylaxis and some patients mislabeled as IA prior to the discovery of α-gal [63]. Interestingly, in a study with 70 patients who were previously diagnosed with IA, a further evaluation showed that 6 of the patients (of whom two had mastocytosis) had indeed reactions related to α-gal sensitization [64•].

In the general adult population, the prevalence of a-gal sensitization ranges between 5.5 and 8.1% in an urban environment [65]. Interestingly, the overall prevalence of α-gal-sIgE-sensitivity was reported to be 20% (≥ 0.10 kU/L) in patients consulting an Allergy Unit, and the highest prevalence (30.2%) was found in patients with insect venom allergies [66•]. Additionally, reported tick bite within the 12 months prior to blood sampling significantly increased the risk of α-gal-sIgE positivity [66•]. Moreover, another study investigated the overall prevalence of α-gal sensitization in patients with clonal MC disorders and found 18%, which appears to be comparable with those of the general population [67]. However, we should keep in mind that not all sensitized patients will experience clinical symptoms; thus, the diagnostic value of α-gal-sIgE needs yet to be clarified.

*Food-dependent exercise-induced anaphylaxis* (FDEIA) should also be considered in differential diagnosis of IA. These reactions may typically begin at any stage of exercise or after just completing exercise, and it is important to know whether or not food ingestion is related to the anaphylactic episodes [68]. Wheat and nuts are the most common culprit foods in Western countries, but shellfish, fruits, vegetables, seeds, legumes, meats, milk, and eggs have all been reported in cases of FDEIA [69–76]. Testing for sIgE to ω-5 gliadin should be considered in patients with suspected wheat-dependent exercise-induced anaphylaxis [77]. The diagnosis often needs to be confirmed with a positive exercise challenge; however, a negative exercise challenge does not exclude FDEIA as illustrated by a literature review of 234 cases of FDEIA patients where food/exercise challenges were performed for 81 patients with 36% of these patients having a negative challenge [78].

### Somatoform Conditions

A group of patients describing subjective symptoms that are consistent with IA without documented objective signs and symptoms have been described and termed to have undifferentiated somatoform IA [79]. Such patients can suffer from panic attacks, vocal cord dysfunction, or Munchausen and can utilize the emergency department repeatedly, for instance, by reporting a sensation of throat tightness (i.e., globus sensation). It may be very challenging to manage these patients, and it is important to evaluate symptoms during acute episodes to confirm whether the reported findings can be documented by a physical examination or an elevation in tryptase or other validated mast cell activation marker during the episode.

## Clinical Vignette

A 68-year-old man without underlying atopies and IgE-mediated food allergies was referred to Allergy Outpatient Clinic at Karolinska Hospital in Stockholm, Sweden, due to 11 unexplained spells attributed to MC activation and MC mediator release. On one occasion, he had an anaphylactic reaction where he had syncope. Patient then became flushed all over the body and had swelling of the wrists. He was sweating before he had diarrhea and fainted shortly after. During other occasions, his reactions followed a typical pattern of itching, flushing, palpitations, abdominal cramps, and diarrhea.

Patient had a baseline tryptase levels of 15 ng/ml. A bone marrow examination was performed but ruled out underlying clonal MC disorders. However, on two of the symptomatic episodes, he sought emergency care and then it was possible to confirm tryptase elevation as the patient had 31 vs. 35 ng/ml, respectively. Thereby, the patient’s symptom patterns and dynamics in his tryptase levels have suggested an underlying systemic mast cell activation process. He was then recommended regular treatment with Desloratadine tablet® (5 mg × 3 times daily) and Montelukast ® (10 mg × 1 daily). Most of the patient’s symptoms had resolved, and during the recent years, he did not experience any severe reaction. Consequently, the diagnosis of idiopathic MCAS was made.

## Management

Although IA episodes can be potentially life-threatening, the frequency and severity of episodes tend to decrease over time [20], as the vast majority of patients have a benign and gradually improving course [80, 81]. Treatment of acute episodes of IA is managed similar to other forms of anaphylaxis, with epinephrine administration being of paramount importance. Evidence suggests that treatment of systemic reactions with epinephrine prevents progression to more severe symptoms [17, 33, 82].

The long-term management for IA aims to reduce the severity and/or frequency of the acute anaphylactic episodes. Nevertheless, evidence for the successful prevention of IA episodes is mainly based on anecdotal case reports for most of the treatment options, as no randomized studies exist to show what treatment(s) are superior in these patients. Hence, we recommend a stepwise approach and management plan to be tailored on a case-by-case basis under close monitoring [33, 50•, 83].

The first step includes H1-histamine receptor antagonists. Doses can be adjusted individually and can be used up to four times similar to those in patients with chronic urticaria. H2-histamine receptor antagonists, antileukotrienes can be additionally given in unresponsive patients. Steroids are typically not required in those with less frequent IA attacks; however, the long-term use of corticosteroids may be necessary in patients with recurrent episodes (> 5/year) [19, 22, 84]. Typical regimens for adults start from 40 to 60 mg prednisone daily and taper the dose by 5 to 10 mg every 2 weeks [19]. In these patients, the lowest dose of steroid capable of preventing anaphylaxis should be considered. The efficacy of the combination of steroids and H1-histamine receptor antagonists can be estimated from case reports and observational series [19, 38]. In the largest published series, 132 of 335 patients were available at the time of data collection [19]. Eighty-seven were in remission, and the duration of remission ranged from 1 to 14 years. Prednisone for frequent episodes had been administered to 56 patients, and approximately 20% had recurrent symptoms as the prednisone was tapered. Among those who eventually weaned off it, the duration of alternate day therapy was 3 months to 13 years.

Steroid-sparing alternatives, including omalizumab, should be considered in patients with recurrent episodes if the combination therapies are ineffective or due to the comorbidity associated with their long-term use. Omalizumab, a monoclonal antibody directed against IgE, has been reported to reduce the frequency of IA episodes in anecdotal reports and case series with varying success [12, 85–87]. However, omalizumab, as all the others, is not a curative therapy and there are currently no randomized, placebo-controlled studies to recommend its routine use.

There are additional therapies that may be beneficial as an “add-on” treatment in some cases. These include ketotifen, and oral cromolyn. Ketotifen is an H1 antihistamine receptor antagonists /mast cell stabilizer that can cause significant sedation. The oral formulation of ketotifen is not approved for use in the USA but the drug can be compounded. Steroid sparing evidence has been published using ketotifen in IA [88–90], and its mast cell stabilizing properties have also been demonstrated in mastocytosis [91, 92]. The usual dose is 1–2 mg orally two or three times per day. Oral cromolyn, which has also local mast cell stabilizing property, can be helpful for patients with prominent gastrointestinal symptoms [19]; however, little is absorbed to systemic circulation, limiting its value as a prophylactic therapy of systemic symptoms. Additionally, some patients do not tolerate this medication because of bloating, cramping, and diarrhea.

## Conclusion

IA is a diagnosis of exclusion and presents with signs and symptoms due to the paroxysmal release of mast cell mediators. During recent years, our understanding of IA has evolved with the recognition of mast cell activation syndromes, mastocytosis, IgE to α-gal or Ω-5-gliadin, and certain medications as causes of anaphylaxis. Management consists of using medications such as antihistamines, sympathomimetic agents, prednisone, and omalizumab. Although there are many unresolved issues regarding the epidemiology and pathogenesis of IA, there are some available evidence to support a mast cell–dependent mechanism which include elevations in tryptase and histamine levels during acute episodes. Thus, these findings support the notion that IA should be considered a form of mast cell activation disorder. Further understanding of the intrinsic changes in mast cells of patients with IA will be an important aspect to address as this area of research continues.
